# Resting-state abnormalities in functional connectivity of the default mode network in migraine: A meta-analysis

**DOI:** 10.3389/fnins.2023.1136790

**Published:** 2023-03-01

**Authors:** Su Hu, Zeqi Hao, Mengting Li, Mengqi Zhao, Jianjie Wen, Yanyan Gao, Qing Wang, Hongyu Xi, Collins Opoku Antwi, Xize Jia, Jun Ren

**Affiliations:** ^1^School of Psychology, Zhejiang Normal University, Jinhua, China; ^2^Key Laboratory of Intelligent Education Technology and Application of Zhejiang Province, Zhejiang Normal University, Jinhua, China; ^3^Department of Radiology, Changshu No.2 People’s Hospital, The Affiliated Changshu Hospital of Xuzhou Medical University, Changshu, Jiangsu, China; ^4^School of Western Languages, Heilongjiang University, Harbin, China

**Keywords:** migraine, meta-analysis, resting-state functional magnetic resonance imaging, default mode network, functional connectivity

## Abstract

Migraine—a disabling neurological disorder, imposes a tremendous burden on societies. To reduce the economic and health toll of the disease, insight into its pathophysiological mechanism is key to improving treatment and prevention. Resting-state functional magnetic resonance imaging (rs-fMRI) studies suggest abnormal functional connectivity (FC) within the default mode network (DMN) in migraine patients. This implies that DMN connectivity change may represent a biomarker for migraine. However, the FC abnormalities appear inconsistent which hinders our understanding of the potential neuropathology. Therefore, we performed a meta-analysis of the FC within the DMN in migraine patients in the resting state to identify the common FC abnormalities. With efficient search and selection strategies, nine studies (published before July, 2022) were retrieved, containing 204 migraine patients and 199 healthy subjects. We meta-analyzed the data using the Anisotropic Effect Size version of Signed Differential Mapping (AES-SDM) method. Compared with healthy subjects, migraine patients showed increased connectivity in the right calcarine gyrus, left inferior occipital gyrus, left postcentral gyrus, right cerebellum, right parahippocampal gyrus, and right posterior cingulate gyrus, while decreased connectivity in the right postcentral gyrus, left superior frontal gyrus, right superior occipital gyrus, right orbital inferior frontal gyrus, left middle occipital gyrus, left middle frontal gyrus and left inferior frontal gyrus. These results provide a new perspective for the study of the pathophysiology of migraine and facilitate a more targeted treatment of migraine in the future.

## 1. Introduction

Migraine is the commonest primary headache in clinical practice. This disabling neurological disorder is characterized by recurrent unilateral or bilateral pulsating headaches (Robbins, 2021). Epidemiological surveys showed that 1.1 billion people suffer from migraine worldwide which constitutes the second leading cause of disability globally (Ashina et al., 2021b; Safiri et al., 2022). Unfortunately, the surveys also illustrated an upward trend in the incidence of migraine attacks (Global Burden of Disease Study 2013 Collaborators, 2015; Burch et al., 2019). Given that the disabling effect of the disease foists colossal socio-economic and health costs on its sufferers (Vo et al., 2018), scholars found the lack of progress in diagnosis and treatment over the years lamentable (Katsarava and Steiner, 2012; Katsarava et al., 2018; Ashina et al., 2021a). We contend that the stagnation in scientific breakthroughs in this area is mainly due to the lack of insight into the disease’s pathophysiology.

Resting-state functional magnetic resonance imaging (rs-fMRI)—a non-invasive method, has been widely used in brain functional research (Yu et al., 2020; Bi et al., 2021). This method has contributed to the understanding of the underlying physiological mechanism of migraine greatly (Colombo et al., 2015; Russo et al., 2017; Ellingson et al., 2019; Kim et al., 2021). Previous rs-fMRI studies demonstrated the close relationship between both the default mode network (DMN) and its associated brain regions and migraine (Jia and Yu, 2017; Chong et al., 2019; Argaman et al., 2020). The DMN is a highly active brain network when the brain is at rest and is associated with an individual’s stressful experiences and his/her coping strategies that promote adaptation to the (stressful) environment (McEwen and Gianaros, 2011; Soares et al., 2013).

A growing body of evidences have revealed the role of non-adaptive responses in migraine mechanisms (Borsook et al., 2012; Maleki et al., 2012). These suggested that recurrent migraine attacks alter the functional connectivity (FC) of the DMN and that these changes may disrupt individual stress response mechanisms (Ellerbrock et al., 2013). Extant studies showed that abnormal FC of DMN has been found in patients with different pain disorders, suggesting that pain has a broad impact on the DMN (Baliki et al., 2008; Napadow et al., 2010). In addition, DMN was involved in the pain inhibition process and affected the efficiency of pain processing (Baliki et al., 2008; Nahman-Averbuch et al., 2014; Youssef et al., 2016). The above studies indicate the critical role DMN plays in the neuropathological mechanisms of migraine. So, a fine understanding of DMN may facilitate workable treatment options.

The identification of spatial patterns of DMN in migraine patients can be based on FC. That is because the FC is capable of exploring the connectivity between brain regions and depicting the complex functional coupling patterns between various brain regions (Biswal et al., 1995), and has been applied in exploring FC abnormalities in patients (Bi et al., 2018). Currently, many scholars have used seed-based FC analysis (Androulakis et al., 2017; Cao et al., 2022) and independent component analysis (Giugni et al., 2010; Xue et al., 2012; Colon et al., 2019) to explore FC abnormalities within the DMN of migraine patients. However, the inconsistent results of the resting-state FC (rs-FC) within the DMN in migraine patients must not be ignored. Some studies identified decreased rs-FC of the DMN in migraine patients compared to healthy subjects. Decreased functional connectivity was found in the right cerebellum and left frontal lobe in Amin et al. (2016) study, Tessitore et al. (2013) found decreased connectivity in the prefrontal and temporal regions of the DMN, Zou et al. (2021) found reduced connectivity in the left precuneus of the DMN while others detected increased rs-FC in the DMN, Chou et al. (2021) observed increased DMN FC in the left precentral gyrus, left postcentral gyrus and right cerebellum, Zhang et al. (2016) only found increased FC in the left precuneus and posterior cingulate cortex within the DMN. Those discrepant findings may be due to small sample size, patients’ clinical heterogeneity, and the different sample selections (Skorobogatykh et al., 2019). Fortunately, meta-analysis can synthesize neuroimaging findings to reduce the discrepant results (Kaiser et al., 2015). Specifically, the method can aggregate and integrate a large amount of data from studies and distinguish positive results in the process of replication (Müller et al., 2018). With the increasing number of published studies about FC in the DMN during the resting state of migraine patients, conducting a meta-analysis to identify common functional abnormalities in the brain is urgently needed to further deepen our understanding of migraine’s pathophysiological mechanisms.

Accordingly, we performed a meta-analysis to assess common alterations in the connectivity of DMN in migraine patients by using the Anisotropic Effect Size version of Signed Differential Mapping (AES-SDM (Radua et al., 2014). Our findings could help harmonize the contradictory results found in the literature and advance the understanding of migraine.

## 2. Methods

### 2.1. Search strategy and literature screening

Methods and analyses of the current study were pre-registered on the PROSPERO (ID: CRD42022348891).^1^ This study was conducted under the Preferred Reporting Items for Rigorous Systematic Reviews and Meta-Analyses (PRISMA) guidelines (Liberati et al., 2009). We searched comprehensively in PubMed, Web of Science, and Embase databases for literature up to July 2022, using the combined keywords: (“migraine”) AND (“resting state” OR “rest”) AND (“DMN” OR “default mode network”). Further, we searched the reference list of the qualified literature compiled by our manual search from related reviews and references. For studies that did not provide detailed information in the paper (e.g., peak effect coordinates), we contacted the authors by email to obtain relevant information.

For literature selection, we used the following inclusion criteria: (i) original rs-fMRI study written in English; (Giugni et al., 2010) patients included in the study meet the diagnostic criteria for migraine; (iii) used seed-based or ICA methods to conduct rs-fMRI data analysis; (iv) direct comparison of rs-FC between migraine patients and healthy subjects; (v) peak coordinates of between-group effects were reported in a standard space, such as Talairach or Montreal Neurological Institute (MNI). Studies were excluded if (i) it is a book chapter or review article; (Giugni et al., 2010) the coordinates of significant statistical differences in rs-FC between groups could not be retrieved even by contacting the author; (iii) the sample in the study overlapped with another published study; and (iv) the seeds were not selected in DMN. According to the above inclusion and exclusion criteria, eligible articles were screened and included in this meta-analysis. The literature search and screening were conducted by two investigators independently (S.H and JJ.W) and the relevant studies were double-checked by the two investigators (S.H and JJ.W). Inconsistent studies were discussed and resolved *via* consensus.

### 2.2. Quality assessment and data extraction

To ensure high data quality, we assessed each included study using a 20-point checklist (Iwabuchi et al., 2015; Pan et al., 2017a), which was adapted from previous rs-fMRI meta-analyses. The checklist mainly contains an assessment of the quality of subjects (e.g., demographic and clinical characteristics) and the study methodology (e.g., image procedure, method description), the details of the checklist are presented in Supplementary Table S1. After quality assessment, important information from each included study was extracted and collated, mainly including the first author, migraine type, sample size, sex ratio, age distribution, disease duration, disease frequency analytical method, seed region, scanner type, and statistical threshold. Furthermore, the current meta-analysis was based on peak coordinates showing the regions of significant differences in rs-FC between-group, therefore, the peak coordinates and their effect values (e.g., *T*-values or *Z*-scores) of each research were also obtained and sorted into a corresponding text file.

### 2.3. Main meta-analysis

An anisotropic effect-size version of seed-based *d* mapping (AES-SDM) software package version 5.15 for Windows^2^ was used in the current meta-analysis. SDM is a powerful statistical technique that uses peak coordinates to perform meta-analyses to assess differences in brain activity and has been widely used in recent neuroimaging meta-analyses (Radua et al., 2012; Lim et al., 2020; Yao et al., 2021). Following the guidance of the AES-SDM tutorial, this meta-analysis was completed in three parts. In the first step, we retrieved the peak coordinates and their corresponding effect size from the results reported by each study. It is worth noting that all *Z*-scores were transformed into *T*-value using the SDM online converter. If the effect size was not specified in the study, we will use “p” for hyper-connectivity and “n” for hypo-connectivity to represent the effect size of these peak coordinates. The peak coordinates and effect values were then saved into a text file. After the data preparation, we preprocessed the datasets and reconstructed a mean effect-size map of differences between groups for each study using an anisotropic unnormalized Gaussian kernel (Radua et al., 2014). In the last step, individual maps from each study were combined using meta-analytic calculations, which were weighted by intra-study variance and inter-study heterogeneity. We then calculated the mean of the study maps with a random-effects model (Radua and Mataix-Cols, 2012; Pan et al., 2017a). Here we adopted the recommended SDM parameters in the main meta-analysis, setting full-width at half-maximum (FWHM) to 20 mm, uncorrected voxel *p* = 0.005, anisotropy = 1, peak height SDM-Z > 1, and cluster extent ≥10 voxels (Radua et al., 2012; Pan et al., 2017c).

### 2.4. Subgroup analysis

In the current study, four types of subgroup analyses were performed. (1) subgroup analyses of migraineurs (MIG) and migraineurs without aura (MowA) to investigate the effect of disease type on FC abnormalities; (2) The five ICA studies were selected as a subgroup and the four studies based on seed-based FC as an additional subgroup to explore the alteration of FC in both methods. (3) To explore the effect of age, a subgroup analysis was performed on adults (eight studies). (4) To control for medication status, we conducted subgroup analyses of whether medication was taken.

### 2.5. Analyses of sensitivity, heterogeneity, and publication bias

A systematic Jackknife sensitivity analysis was conducted to test the robustness and reliability of the main results. In short, Jackknife sensitivity analysis consists of repeated analyses, discarding one study at a time, for assessing the reproduction of the results (Radua et al., 2012). In this analysis, we used the “leave-one-out cross-validation” (LOOCV) method to verify the repeatability of the results (Radua and Mataix-Cols, 2009). To be specific, the main analysis was repeated n times (*n* = the number of total studies), while discarding a different study each time. If the significant brain areas obtained after each LOOCV analysis were present in all or most of the previous significant brain regions, this result is then considered stable and reproducible (Duko et al., 2020). Similarly, we applied the default SDM kernel size and thresholds to obtain the heterogeneous brain regions (Radua et al., 2014), identifying brain areas that were not explained by differences between studies by using random effects models and Q test, H test, I-square statistics (Iwabuchi et al., 2015; Ueno et al., 2016). Heterogeneity in the present study was evaluated by converting Q-statistics to Z-scores. Clusters showing significant heterogeneity and overlapped with the primary outcome were considered to be between-study heterogeneity (Viechtbauer, 2005; Albajes-Eizagirre and Radua, 2018). The heterogeneous brain regions obtained cannot be interpreted simply from the cause of the disease; it may result from clinical differences or demographic information of the patients, so the interpretation of the heterogeneous brain regions needs to be done with caution. Specifically, the results were thresholded using FWHM = 20 mm, uncorrected *p* = 0.005, peak height *Z* > 1, and cluster extent ≥10 voxels (Pan et al., 2017c; Zhen et al., 2018). In addition, funnel plots and Egger’s test in SDM were conducted to calculate the probability of publication bias. Publication bias is the tendency for statistically significant findings (positive results) to be reported and published more frequently than non-significant findings (negative results) or invalid findings (Egger et al., 1997). And the Egger’s test was conducted by extracting values from the peak coordinates of abnormal brain regions in the main meta-analysis results (Zhen et al., 2018). Specifically, we first created a mask that using the peak coordinate from the main meta-analysis results, and then extracted value within mask. A result of *p* < 0.05 in Egger’s test was considered to reflect significant publication bias (Pan et al., 2017b; Zhen et al., 2018).

### 2.6. Meta-regression analysis

The meta-regression analysis was conducted to examine the potential effects of the relevant variables on the FC alteration, in which the main variables involved were mainly age, disease duration, and frequency of attack. For the reduction of spurious relations, the significance level was set at *p* < 0.0005 as well as an extent threshold of 100 voxels (Radua and Mataix-Cols, 2009).

## 3. Results

### 3.1. Included studies and sample characteristics

**FIGURE 1:**
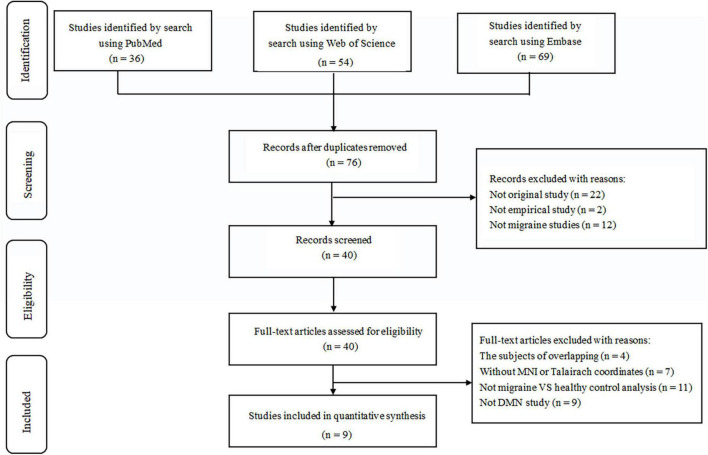
Flow diagram for the identification and exclusion of studies. MNI, Montreal Neurological Institute; DMN, default mode network.

Following strict inclusion and exclusion criteria, we found nine studies that met our requirements and consisted of 204 migraine patients and 199 healthy individuals. The flow chart for the search and selection strategies are shown in Figure 1. One of the studies didn’t report the sex ratio and another did not report age distribution. In the nine included studies, patients in three studies with medication status. In Hubbard et al. (2014) study, the majority of migraine took prophylactic medication, whereas over a third of migraine took abortive medications in Lo Buono et al. (2017) study, patients took simple analgesics, simple triptans, and combination analgesics during a migraine attack; in Messina et al. (2020) study, patients were taking flunarizine and ginkgolide for migraine prevention at the time of MRI. All the studies were acquired using a 3.0 T MRI scanner. There are five studies for ICA analysis and four studies for seed-based rs-FC analysis. The detailed sample characteristics and methodological details of included studies are summarized in Table 1.

**TABLE 1:** Characteristics of resting-state functional magnetic resonance imaging (rs-fMRI) studies included in the meta-analysis.

References	Migraine subtype	Subjects (female)		Age (mean ± SD/SE)		Method of analysis	Seed region	Disease duration (year)	Disease frequency (times/months)	Medication (on/off)	Scanner (Tesla)	Statistical threshold	Quality scores (out of 20)
		Patients	HCs	Patients	HCs								
Cao et al., 2022	MwoA-DI	34 (25)	44 (33)	34.44 ± 10.04	30.63 ± 9.56	Seed-based analysis	The left middle frontal cortex (lower)^1^	10.68 ± 10.03	9.61 ± 8.98	Off	3.0 T	FWE (*p* < 0.001)	18
Chou et al., 2021	MIG	50 (44)	30 (23)	40.14 ± 8.83	41.47 ± 10.41	Seed-based analysis	DMPFC, PMC, TPJ	15.63 ± 10.55	7.38 ± 5.58	Off	3.0 T	FWE (*p* < 0.05)	19
Colon et al., 2019	MIG	18 (9)	18 (9)	23.22 ± 2.07	22.91 ± 2.08	ICA	–	9.56 ± 3.87	2.8 ± 2.57	Off	3.0 T	NA	19
Hubbard et al., 2014	MIG	17 (13)	18 (14)	41.71 ± 12.20	38.89 ± 11.25	Seed-based analysis	L PCC^2^	NA	NA	On	3.0 T	FWE (*p* < 0.005)	19
Lo Buono et al., 2017	MwoA	14 (NA)	14 (NA)	40.75 ± 11.82	41.75 ± 12.82	ICA	–	12.3 ± 5.8	6.07 ± 2.81	On	3.0 T	Permutation (*p* < 0.05)	17
Messina et al., 2020	MIG	13 (7)	14 (6)	13.8 (NA)	13.6 (NA)	ICA	–	NA	NA	On	3.0 T	FWE (*p* < 0.05)	17
Tessitore et al., 2013	MwoA	20 (10)	20 (10)	28.15 ± 3.08 (SE)	28.90 ± 3.63 (SE)	ICA	–	8.22 ± 2.04	6 ± 2.04	Off	3.0 T	FDR (*p* < 0.05)	19
Zhang et al., 2016	MwoA	22 (13)	22 (13)	41.8 ± 10.2	42.0 ± 10.3	Seed-based analysis	Precuneus^3^	9.8 ± 7.3	3.2 ± 2.2	Off	3.0 T	FDR (*p* < 0.005)	20
Zou et al., 2021	CM	17 (10)	20 (11)	45.41 ± 14.87	43.56 ± 8.77	ICA	–	10.47 ± 3.99	10.65 ± 2.83	Off	3.0 T	AlphaSim (*p* < 0.05)	20

HCs, healthy controls; ICA, Independent Component Analysis; SD, standard deviation; SE, standard error; MwoA-DI, migraine without aura present during the interictal period; MIG, migraine; MwoA, migraine without aura; CM, chronic migraine; DMPFC, dorsomedial prefrontal cortex; PCC, posterior cingulate cortex; PMC, posteromedial cortex; LTPJ, left temporoparietal junctions; FWE, family wise error; FDR, false discovery rate; NA, not available. Montreal Neurological Institute (MNI) coordinates (if available): 1 (–42, 57, 12), 2 (–8, –46, 27), 3 (–6, –54, 18). “1,2,3” represent the coordinates of the three seed regions.

### 3.2. Main meta-analysis of rs-FC between migraine and HCs

**FIGURE 2:**
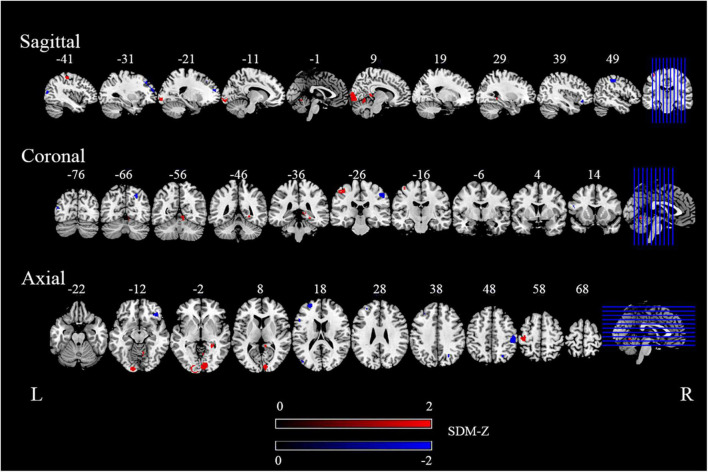
The areas of increased (red) and decreased (blue) functional connectivity (FC) in the main meta-analysis. “R” and “L” denote the right and left sides of the brain, respectively. The color bar indicates the maximum and minimum seed-based d mapping (SDM)-Z value.

Compared with the HCs, we found migraine patients to show significantly increased rs-FC, including right calcarine gyrus (Calcarine_R), left inferior occipital gyrus (Occipital_Inf_L), left postcentral gyrus (Postcentral_L), right cerebellum (lobule IV/V, Cerebelum_4_5_R), right parahippocampal gyrus (ParaHippocampal_R), and right posterior cingulate gyrus (Cingulum_Post_R). Meanwhile, decreased rs-FC were discovered in the right postcentral gyrus (Postcentral_R), left superior frontal gyrus (Frontal_Sup_L), right superior occipital gyrus (Occipital_Sup_R), right orbital inferior frontal gyrus (Frontal_Inf_Orb_R), left middle occipital gyrus (Occipital_Mid_L), left middle frontal gyrus (Frontal_Mid_L), and left inferior frontal gyrus triangular part (Frontal_Inf_Tri_L). The detailed results from the meta-analysis are summarized in Figure 2, Table 2.

**TABLE 2:** Clusters showing resting-state functional connectivity (rs-FC) differences in migraine patients compared with healthy control (HC).

Location	MNI coordinate			Cluster size	SDM-Z value	Effect size	*P*-value	Jacknife sensitivity analysis	Heterogeneity	Egger test (*p*-value)
	x	y	z							
**Migraine > HC**										
Calcarine_R (aal)	8	–90	10	430	1.685	0.171	≤0.001	9/9	Yes	0.652
Occipital_Inf_L (aal)	–20	–94	–10	249	1.628	0.165	0.001	7/9	No	0.629
Postcentral_L (aal)	–40	–26	56	152	1.692	0.173	≤0.001	7/9	No	0.343
Cerebelum_4_5_R (aal)	8	–56	–4	70	1.584	0.160	0.001	8/9	No	0.294
ParaHippocampal_R (aal)	24	–42	–4	58	1.615	0.158	0.001	7/9	No	0.437
Cingulum_Post_R (aal)	10	–42	8	57	1.670	0.169	0.001	8/9	No	0.294
**Migraine < HC**										
Postcentral_R (aal)	50	–30	46	218	–1.451	–0.200	0.002	7/9	No	0.363
Frontal_Sup_L (aal)	–26	52	20	106	–1.467	–0.206	0.002	7/9	No	0.395
Occipital_Sup_R (aal)	26	–66	44	78	–1.425	–0.182	0.002	6/9	No	0.392
Frontal_Inf_Orb_R (aal)	40	32	–10	71	–1.404	–0.167	0.003	6/9	No	0.401
Occipital_Mid_L (aal)	–42	–80	12	70	–1.427	–0.210	0.002	6/9	No	0.188
Frontal_Mid_L (aal)	–30	30	42	41	–1.574	–0.159	0.001	9/9	No	0.140
Frontal_Inf_Tri_L (aal)	–50	18	16	21	–1.389	–0.159	0.003	6/9	No	0.416

aal, automated anatomical labeling; HC, healthy controls; MNI, Montreal Neurological Institute; SDM, seed-based d mapping; Calcarine_R, right calcarine gyrus; Occipital_Inf_L, left inferior occipital gyrus; Postcentral_L, left postcentral gyrus; Cerebelum_4_5_R, right cerebellum (lobules IV/V); ParaHippocampal_R, right parahippocampal gyrus; Cingulum_Post_R, right posterior cingulate gyrus; Postcentral_R, right postcentral gyrus; Frontal_Sup_L, left superior frontal gyrus; Occipital_Sup_R, right superior occipital gyrus; Frontal_Inf_Orb_R, right orbital inferior frontal gyrus; Occipital_Mid_L, left middle occipital gyrus; Frontal_Mid_L, left middle frontal gyrus; Frontal_Inf_Tri_L, left inferior frontal gyrus triangular part.

### 3.3. Subgroup meta-analysis

Subgroup analyses of ICA studies suggested that decreased FC was still found in the Occipital_Mid_L, Frontal_Sup_L and we also found decreased FC in the Calcarine_R in patients compared with healthy subjects. While the increased FC showed some subtle difference. Increased FC was found in the right cuneus gyrus (Cuneus_R) and the left temporal pole of superior temporal gyrus (Temporal_Pole_Sup_L) (Supplementary Table S2 and Supplementary Figure S1). Subgroup analysis of seed-based FC revealed that decreased FC was still found in the Occipital_Sup_R, the Frontal_Mid_L, and the Frontal_Inf_Orb_L. At the same time, we also found increased FC in cerebellum, such as right Crus I and II of cerebellar hemisphere gyrus. Besides, we found decreased FC in the right supramarginal gyrus (SupraMarginal_R), the inferior frontal gyrus of orbital part (Frontal_Inf_Orb) while increased FC was found in the right gyrus rectus (Rectus_R), right lingual gyrus (Lingual_R) and lobule of vermis (Supplementary Table S3 and Supplementary Figure S2). For the migraine without aura (MwoA), increased FC was revealed in left lingual gyrus (Lingual_L), the left superior temporal gyrus (Temporal_Pole_Sup_L), right calcarine gyrus (Calcarine_R), and left superior frontal gyrus (Frontal_Sup_L), while decreased FC was found right median cingulate and paracingulate gyri (Cingulum_Mid_R), Frontal_Mid_L, and right precuneus (Precuneus_R) (Supplementary Table S2 and Supplementary Figure S1); About migraine (MIG) group, we also found increased FC in the Postcentral_L, decreased FC in Frontal_Inf_Tri_L and Frontal_Sup_L, which is consistent with the main results. Besides, we found some different brain region in MIG group, the left temporal pole of superior temporal gyrus (Temporal_Pole_Sup_L), the left precentral gyrus (Precentral_L), the left superior parietal gyrus (Parietal_Sup_L), and the left precuneus gyrus (Precuneus_L) was increase in FC compared with healthy subjects, while the left inferior parietal (Parietal_Inf_L) and the left precuneus was decrease in FC relative to healthy subjects (Supplementary Table S5 and Supplementary Figure S4). For the adult subgroup meta-analysis, the great majority of the results were consistent with the main meta-analysis results. However, the increased FC was also found in the Precuneus_R and Lingual_R, the decreased FC was also showed in the right supramarginal gyrus (SurpraMarginal_R) and the left inferior frontal gyrus of opercular part (Frontal_Inf_Oper_L) in adult group (Supplementary Table S6 and Supplementary Figure S5). For the no-medication subgroup analysis, decreased FC in the Frontal_Mid_L and Frontal_Sup_L was also found, while increased FC was showed in the precentral gyrus (Precentral_L) and left middle temporal gyrus (Temporal_Mid_L) in migraine relative to healthy subjects. About subgroup analyses of medication status, Clusters still showing increase FC in the calcarine gyrus, decreased in Postcentral_R, Occipital_Sup_R, Frontal_Inf_Orb_R in migraine patients compared with healthy subjects (Supplementary Tables S7, S8 and Supplementary Figures S6, S7).

### 3.4. Sensitivity test for the main findings

Jackknife sensitivity analysis revealed that rs-FC results remained largely consistent. After nine repeated meta-analyses using the “leave-one-out cross-validation” (LOOCV), as shown in Table 2, the Calcarine_R and Frontal_Mid_L were replicated in all nine meta-analyses. The right cerebellum and Cingulum_Post_R were stably preserved in 8/9 of the datasets. The results in the Occipital_Inf_L, Postcentral_L, ParaHippocampal_R, Postcentral_R, and Frontal_Sup_L remained significant in seven meta-analyses. Other brain regions remained significant in six meta-analyses. The heterogeneity analysis revealed significant between-study variability of FC changes in the right calcarine gyrus; this may have arisen due to the clinical differences between studies (Sterne et al., 2011). In addition, none of the brain regions showed significant publication bias based on Egger’s test (*p* > 0.05). And we provided funnel plots of the meta-analysis to visualize the possibility of publication bias in Supplementary Figures S8–S11.

### 3.5. Meta-regression analysis

Age, the duration of the disease and the frequency of attacks of patients in included studies were collated by us in current study. In the meta-regression analysis, no significant correlation was found between the mean age of the patients (available in all studies), the duration of the disease [available in all studies but two (Hubbard et al., 2014; Messina et al., 2020)] or attack frequency [available in all studies but two (Hubbard et al., 2014; Messina et al., 2020)] and the abnormality of FC.

## 4. Discussion

This meta-analysis was the first study to systematically examine the rs-FC within DMN in migraine patients. The current study comprehensively reviewed nine studies, using a quantitative SDM meta-analysis to reveal abnormalities associated with DMN in migraine. We found that compared with healthy subjects, migraine patients showed abnormal connectivity of rs-FC in key nodes of DMN, such as ParaHippocampal_R, Frontal_Mid_L, Cingulum_Post_R. In addition to this, there were abnormalities of rs-FC presented in other cortical areas, like the occipital lobe, cerebellum, and some region of the injury perception pathway. The present study highlighted the crucial role of the DMN in migraine pathology, and provides a reliable reference for further understanding of its pathogenesis.

In specific terms, we found reduced FC in the Frontal_Mid_L, Frontal_Sup_L, and Frontal_Inf_Tri_L, Frontal_Mid_L as part of the prefrontal cortex, was engaged in the cognitive assessment and regulation of pain (Katja et al., 2008). In a prior functional brain pathway study, FC of the middle frontal gyrus and the dorsal anterior cingulate cortex were reduced in migraine patients without aura compared to healthy subjects (Russo et al., 2012). This reduced connectivity of the middle frontal gyrus was negatively correlated with the pain intensity of migraine attack (Russo et al., 2012). Brain structure in migraine patients revealed that the gray matter volume and the folding coefficient of the middle frontal gyrus were reduced in migraine patients without aura (Jia and Yu, 2017). The superior frontal gyrus was involved in emotional responses and the participative feelings of pain (Bluhm et al., 2007). The left inferior frontal gyrus played a role in cognitive modulation of pain as well as memory retrieval and emotional pain regulation (Ochsner et al., 2012). Several studies supported linking migraine physiopathology with the left inferior frontal gyrus (Arkink et al., 2012; Zhang et al., 2020). For example, aberrant cerebral perfusion changes in the left inferior frontal gyrus in migraineurs have been found (Arkink et al., 2012). In our subgroup analysis, we also found abnormalities of FC in other brain regions (inferior frontal gyrus of orbital part and opercular part) of the frontal lobe. These brain regions of rs-FC abnormalities were associated with migraine and other pain disorders in the past studies (Xu et al., 2022; Yang et al., 2022), indirectly suggesting that the frontal cortex plays an important role in pain perception and regulation, and to a certain extent, it is possibly involved in the development of migraine.

In the current study, the right calcarine gyrus (Calcarine_R), superior occipital gyrus (Occipital_Sup_R), left middle occipital gyrus (Occipital_Mid_L), and inferior occipital gyrus (Occipital_Inf_L), which are part of the occipital lobe, showed abnormal FC in migraine patients. Abnormalities in the occipital lobe were found to closely relate to migraine (Schoenen et al., 2003; Vincent and Hadjikhani, 2007a). For example, the theory of cortical spreading of depression is an important hypothesis of migraine and is widely considered to be the pathological mechanism of migraine with aura, and emphasizes the importance of abnormalities in the occipital lobe in migraine (Hadjikhani et al., 2001; Tfelt-Hansen, 2010). Besides, the occipital lobe is now generally considered to be hyperexcitable in migraine (Welch, 2005). Such excitability leads to a lower threshold for migraine attacks, and that cortical diffusion inhibition arises when certain stimuli are present, leading to migraine attacks. In other words, when patients suffer migraine attacks, the body’s self-protective mechanisms may reduce the frequency and the degree of pain of migraine attacks by decreasing the excitability of the occipital cortex. Consistent with these studies, we also found abnormal FC in brain regions associated with the occipital cortex. This reflected the idea that the occipital lobe related to visual function may be an important pathogenesis of migraine. Besides, we also found abnormal FC in the cuneus and lingual gyrus in some subgroup analyses, these regions are the major regions of the visual network in previous studies (Boulloche et al., 2010; Lee et al., 2019). Established studies illustrated the hypermetabolism of the lingual gyrus and the cuneus, which is involved in the perceptual abnormalities associated with visual, such as photophobia (Denuelle et al., 2011; Schankin et al., 2014). It is necessary to note that Calcarine_R is heterogeneous in the results, which may be due to clinical variations in patients between studies (Sterne et al., 2011). Hence this abnormality in FC of this brain region needs to be treated with caution and suggests that we can pay further attention to abnormal variations in Calcarine_R in migraine patients in the future.

Our meta-analysis also revealed enhanced rs-FC in the right posterior cingulate gyrus (Cingulum_Post_R) and the right parahippocampal gyrus (ParaHippocampal_R) in migraine patients. Migraine patients often suffer from pain. While the pain processing is complex and it may be regulated by brain regions of DMN, such as the cingulate gyrus and ParaHippocampal_R (Tomasi and Volkow, 2011). For example, the ParaHippocampal_R participates in the abnormal processing of allodynia (Iadarola et al., 1998). Metabolic abnormalities in the posterior cingulate gyrus have been suggested in previous studies in migraine patients without aura (Kim et al., 2010), and a significant negative correlation between the gray matter density of the posterior cingulate gyrus bilaterally and pain sensitivity was also noted (Emerson et al., 2014). The present results imply that these regions may be involved in pain modulation for migraine patients.

Migraine patients also showed FC abnormalities on the postcentral gyrus in the main results and abnormalities on the precentral gyrus in the subgroup analyses compared to healthy subjects. The postcentral gyrus precentral gyrus is not only a key brain region in the central brain network of pain modulation responsible for information processing of painful stimuli (Apkarian et al., 2005; Schweizer et al., 2008; Tracey, 2008; Torta et al., 2017), it has also been suggested to be involved in the trigeminal-thalamo-cortical injury perception pathway, which is related to the pathophysiology of migraine (Goadsby et al., 2017). For example, migraine patients demonstrated significantly decreased FC between the right postcentral gyrus and right substantia nigra, and the altered FC was negatively associated with migraine duration (Huang et al., 2019). Meanwhile, the reduced low-frequency amplitude of the postcentral gyrus in patients was captured in the neural activity during the migraine interval (Wang et al., 2016) and cortical thinning within the postcentral gyrus in migraine patients (Mehnert et al., 2017). Consistent with the results of these studies, the present study also found a decrease in FC of postcentral gyrus in migraine patients. Additionally, MRI studies showed that the postcentral gyrus is activated by painful stimulation (Carlsson et al., 2006; Wager et al., 2013). Increase in FC also has been found in the left postcentral gyrus in this study, this may be due to subtle differences in brain function between the left and right hemispheres. Therefore, it is necessary to explore the abnormalities of the left and right postcentral gyrus in migraine patients in the future. Abnormalities of the postcentral gyrus in patients may be associated with disruptions in networks related to pain modulation. This further suggests that the postcentral gyrus in the “central brain network of pain modulation” may be characteristically altered in migraine patients.

Enhanced FC was found in the right cerebellum (lobule IV/V) in this study, which is consistent with the results of previous studies. The cerebellum is primarily responsible for nociceptive avoidance behavior. For instance, when there is an increased spontaneous cerebellar activity, the cerebellum has stronger resistance to injury (Pereira et al., 2017). Some studies have found that the cerebellum plays a regulatory role in pain processing and pain perception (Ruscheweyh et al., 2014; Mehnert et al., 2017). Established studies provided increasing evidence that the cerebellum is associated with migraine. For example, studies have found abnormal cerebellar function in migraine patients, mainly in terms of cerebellar hyperactivation and abnormal spontaneous cerebellar activity when stimulated by negative emotional images (Wang et al., 2016, 2017). In terms of structure, it has also been found that the gray matter volume of cerebellar regions is smaller in patients with chronic migraine compared to healthy subjects (Bilgiç et al., 2016; Lai et al., 2016). The present results further support the potential association of the cerebellum with the pathogenesis of migraine (Vincent and Hadjikhani, 2007b).

In additional, influenced by different methods, disease subtypes and medication use, we also found abnormalities of FC in other brain regions. The temporal lobe (temporal pole of superior temporal gyrus, middle temporal gyrus and inferior temporal gyrus) is significantly affected by migraine in our analyses, which is consistent with the results of previous studies (Afridi et al., 2005). And the temporal lobe is an associative multisensory area that also processes visual and auditory information have been confirmed (Moulton et al., 2011). The precuneus is a core region of the DMN involved in migraine in our subgroup analyses. Abnormalities in the precuneus have been found to potentially affect information transmission, multimodal integration, and pain sensitivity and processing in patients with MwoA (Zhang et al., 2016; Li et al., 2022). Abnormalities in FC of the supramarginal gyrus have also been reported frequently in subgroup analyses. The supramarginal gyrus is particularly engaged in the cognitive assessment of pain (Lamm et al., 2011; Moulton et al., 2012), and reduced pain-related activity in the suprachiasmatic gyrus has been reported in patients with overmedicated headaches (Ferraro et al., 2012). The results of these subgroup analyses suggested that FC abnormalities between patients with different clinical information could be studied in detail to more precisely understand the pathogenesis of migraine.

Several limitations need to be considered in this study. Firstly, the nine studies included in our meta-analysis differ in age and gender of the subjects, and migraine patients of different genders and age stages may exhibit different DMN abnormalities. We recommend that future studies systematically analyze data from the same age level or the same gender population. Secondly, the AES-SDM approach used in the present study is based on the reported coordinates of previous studies rather than the raw imaging data, future studies could conduct meta-analyses based on the brain maps to add more detailed information. Finally, despite our efforts to obtain information, the number of included studies was relatively small (i.e., nine studies), further research in this field is necessary to confirm and extend these findings.

## 5. Conclusion

In the current study, we included all available publications using an rs-FC analysis within DMN and performed a meta-analysis to determine the consistency and robustness of altered FC within the DMN in migraine patients. Migraine patients exhibited abnormal FC within DMN as well as some other regions associated with the central brain network of pain modulation. The present study can be considered an exploratory study that provides the primary evidence for the study of functional abnormalities in the DMN in migraine patients. The findings of this study improved our understanding of the pathophysiological mechanisms of migraine from a systemic perspective.

## Author contributions

SH, XJ, and JR contributed to the conception of the study. ML, MZ, JW, YG, and QW performed the experiment and analysis preparation. SH and ZH wrote the first manuscript. HX and CA helped coordinate the study and reviewed the manuscript. All authors read and approved the final manuscript.

## Footnotes

1
https://www.crd.york.ac.uk/prospero/#searchadvanced

2
https://www.sdmproject.com/software/
