# Supplementary material for: Resting-state abnormalities in functional connectivity of the default mode network in migraine: A meta-analysis
Source: Front Neurosci. 2023 Mar 1;17:1136790. doi: 10.3389/fnins.2023.1136790 (PMC10014826; doi:10.3389/fnins.2023.1136790)
Supplement: Supplementary file 1 [file Data_Sheet_1.ZIP › Supplementary materials.docx]

Supplementary Material

**Supplementary Tables and Figures**

Table S1: Criteria for objective assessment of methodological quality of individual studies.

| **Category 1: Sample characteristics (10)** |
| --- |
| Patients were evaluated with specific standardized diagnostic criteria (1) |
| Important demographic data (age and gender) were reported with mean (or median) and standard deviations (or range)) (2) |
| Healthy comparison subjects were evaluated to exclude psychiatric and medical illnesses and demographic data was reported (1) |
| Important clinical variables (e.g. illness duration, medication status, HAMA scores, HAMD scores) were reported with mean (or median) and standard deviations (or range)) (4) |
| Sample size per group > 10 (2) |
| **Category 2: Methodology and reporting (10)** |
| Whole brain analysis was automated with no a-priori regional selection (3) |
| Magnet strength at least 1.5T (1) |
| At least 5 minutes of resting state acquisition (1) |
| Whole brain coverage of resting scans (1) |
| The acquisition and preprocessing techniques were clearly described so that they could be reproduced (1) |
| Coordinates reported in a standard space (1) |
| Significant results are reported after correction for multiple testing using a standard statistical procedure (FDR, FWE or permutation-based methods) (1) |
| Conclusions were consistent with the results obtained and the limitations were discussed (1) |

A maximum score of 20 for each study, allocated as per the criteria specified above. Each of the two sections, sample characteristics, methodology and reporting, is worth 10 points, and the value following each item represents the score that the item would have received if it had met the requirements. Take the first project as an example, patients were assessed in a study using specific standardized diagnostic criteria, then the study would receive a score of 1.

Table S2: Clusters showing rs-FC differences in migraine patients compared with HC in subgroup analysis of ICA method.

| Location | MNI coordinate | | | Cluster  size | SDM-Z  value | Effect size | *p* value |
| --- | --- | --- | --- | --- | --- | --- | --- |
|  | x | y | z |  |  |  |  |
| Migraine > HC |  |  |  |  |  |  |  |
| Cuneus_R (aal) | 10 | -90 | 16 | 493 | 1.533 | 0.252 | 0.000456 |
| Temporal_Pole_Sup_L (aal) | -26 | 6 | -26 | 236 | 1.079 | 0.168 | 0.003416 |
| Migraine < HC |  |  |  |  |  |  |  |
| Occipital_Mid_L (aal) | -38 | -78 | 24 | 381 | -1.089 | -0.363 | 0.000233 |
| Frontal_Sup_L (aal) | -18 | 60 | 16 | 171 | -1.018 | -0.159 | 0.000769 |
| Calcarine_R (aal) | 6 | -64 | 14 | 31 | -1.018 | -0.159 | 0.000769 |

Abbreviation: aal, automated anatomical labeling; HC, healthy controls; MNI, Montreal Neurological Institute; SDM, seed-based d mapping; Cuneus_R, right cuneus gyrus; Temporal_Pole_Sup_L, left temporal pole: superior temporal gyrus; Frontal_Sup_L, left superior frontal gyrus, dorsolateral; Occipital_Mid_L, left middle occipital gyrus; Frontal_Sup_L, left superior frontal gyrus, dorsolateral; Calcarine_R, right calcarine fissure and surrounding cortex.

Table S3: Clusters showing rs-FC differences in migraine patients compared with HC in subgroup analysis of seed-based function connectivity method.

| Location | MNI coordinate | | | Cluster  size | SDM-Z  value | Effect size | *p* value |
| --- | --- | --- | --- | --- | --- | --- | --- |
|  | x | y | z |  |  |  |  |
| Migraine > HC |  |  |  |  |  |  |  |
| Vermis_4_5 (aal) | 2 | -56 | 2 | 534 | 1.760 | 0.238 | 0.000003 |
| Cerebellum_Crus1_R (aal) | 32 | -64 | -28 | 22 | 1.185 | 0.157 | 0.002546 |
| Rectus_R (aal) | 4 | 36 | -20 | 17 | 1.185 | 0.157 | 0.002546 |
| Cerebellum_Crus1_R (aal) | 20 | -78 | -32 | 17 | 1.184 | 0.157 | 0.002546 |
| Lingual_R (aal) | 14 | -82 | -12 | 10 | 1.184 | 0.157 | 0.002778 |
| Cerebellum_Crus2_R (aal) | 38 | -72 | -40 | 10 | 1.184 | 0.157 | 0.002546 |
| Vermis_6 | -2 | -72 | -14 | 10 | 1.183 | 0.157 | 0.002989 |
| Migraine < HC |  |  |  |  |  |  |  |
| SupraMarginal_R (aal) | 50 | -30 | 46 | 248 | -1.617 | -0.445 | 0.001967 |
| Occipital_Sup_R (aal) | 26 | -66 | 44 | 112 | -1.585 | -0.403 | 0.002402 |
| Frontal_Mid_L (aal) | -30 | 36 | 38 | 98 | -1.789 | -0.238 | 0.000517 |
| Frontal_Inf_Orb_R (aal) | 40 | 32 | -10 | 92 | -1.564 | -0.370 | 0.0027037 |
| Frontal_Inf_Orb_L (aal) | -50 | 16 | 18 | 33 | -1.548 | -0.367 | 0.0027037 |

Abbreviation: aal, automated anatomical labeling; HC, healthy controls; MNI, Montreal Neurological Institute; SDM, seed-based d mapping; Vermis_4_5, lobule IV, V of vermis; Cerebelum_Crus1_R, right Crus I of cerebellar hemisphere gyrus; Rectus_R, right gyrus rectus ; Lingual_R, right Lingual gyrus; Cerebelum_Crus2_R, right crus II of cerebellar hemisphere; Vermis_6, lobule VI of vermis; SupraMarginal_R, right supramarginal gyrus; Occipital_Sup_R;, right superior occipital gyrus; Frontal_Mid_L, left middle frontal gyrus; Frontal_Inf_Orb_R, right inferior frontal gyrus, orbital part; Frontal_Inf_Orb_L, left inferior frontal gyrus, orbital part.

Table S4: Clusters showing rs-FC differences in migraine patients compared with HC in subgroup analysis of migraine without aura (MowA).

| Location | MNI coordinate | | | Cluster  size | SDM-Z  value | Effect size | *p* value |
| --- | --- | --- | --- | --- | --- | --- | --- |
|  | x | y | z |  |  |  |  |
| Migraine > HC |  |  |  |  |  |  |  |
| Lingual_L (aal) | -10 | -48 | 4 | 883 | 1.569 | 0.401 | 0.000086 |
| Temporal_Pole_Sup_L (aal) | -34 | 10 | -18 | 232 | 1.028 | 0.326 | 0.002081 |
| Calcarine_R (aal) | 10 | -94 | 2 | 100 | 1.079 | 0.335 | 0.001603 |
| Frontal_Sup_L (aal) | -14 | 50 | 40 | 65 | 1.027 | 0.324 | 0.002114 |
| Migraine < HC |  |  |  |  |  |  |  |
| Cingulum_Mid_R (aal) | 4 | -38 | 42 | 820 | -2.261 | -0.333 | 0.000003 |
| Frontal_Mid_L (aal) | -32 | 32 | 40 | 66 | -2.012 | -0.296 | 0.000719 |
| Precuneus_R (aal) | 6 | -62 | 38 | 43 | -1.704 | -0.250 | 0.002828 |

Abbreviation: aal, automated anatomical labeling; HC, healthy controls; MNI, Montreal Neurological Institute; SDM, seed-based d mapping; Lingual_L, left lingual gyrus; Temporal_Pole_Sup_L, left temporal pole: superior temporal gyrus; Calcarine_R, right calcarine fissure and surrounding cortex; Frontal_Sup_L, left superior frontal gyrus, dorsolateral; Cingulum_Mid_R, right median cingulate and paracingulate gyri; Frontal_Mid_L, left middle frontal gyrus; Precuneus_R, right precuneus.

Table S5: Clusters showing rs-FC differences in migraine patients compared with HC in subgroup analysis of migraine (MIG).

| Location | MNI coordinate | | | Cluster  size | SDM-Z  value | Effect size | *p* value |
| --- | --- | --- | --- | --- | --- | --- | --- |
|  | x | y | z |  |  |  |  |
| Migraine > HC |  |  |  |  |  |  |  |
| Temporal_Pole_Sup_L (aal) | -46 | 8 | -14 | 334 | 2.015 | 0.273 | 0.000111 |
| Postcentral_L (aal) | -38 | -24 | 52 | 271 | 1.943 | 0.265 | 0.000246 |
| Precentral_L (aal) | -22 | -20 | 64 | 38 | 1.850 | 0.250 | 0.000757 |
| Parietal_Sup_L (aal) | -14 | -68 | 50 | 25 | 1.702 | 0.230 | 0.002477 |
| Precuneus_L (aal) | -12 | -54 | 30 | 14 | 1.764 | 0.239 | 0.001612 |
| Migraine < HC |  |  |  |  |  |  |  |
| Frontal_Inf_Tri_L (aal) | -52 | 16 | 28 | 278 | -1.048 | -0.285 | 0.000961 |
| Frontal_Sup_L (aal) | -20 | 54 | 16 | 65 | -1.018 | -0.252 | 0.001424 |
| Parietal_Inf_L (aal) | -32 | -38 | 42 | 61 | -1.075 | -0.318 | 0.000789 |
| Precuneus_L (aal) | -2 | -54 | 12 | 38 | -1.014 | -0.247 | 0.001472 |

Abbreviation: aal, automated anatomical labeling; HC, healthy controls; MNI, Montreal Neurological Institute; SDM, seed-based d mapping; Temporal_Pole_Sup_L, left temporal pole: superior temporal gyrus; Postcentral_L, left postcentral gyrus; Precentral_L, left precentral gyrus; Parietal_Sup_L, left superior parietal gyrus; Precuneus_L, left precuneus gyrus; Frontal_Inf_Tri_L, left inferior frontal gyrus, triangular part; Frontal_Sup_L, left superior frontal gyrus, dorsolateral; Parietal_Inf_L, left inferior parietal, but supramarginal and angular gyri; Precuneus_L, left precuneus.

Table S6: Clusters showing rs-FC differences in migraine patients compared with HC in subgroup analysis of adults group.

| Location | MNI coordinate | | | Cluster  size | SDM-Z  value | Effect size | *p* value |
| --- | --- | --- | --- | --- | --- | --- | --- |
|  | x | y | z |  |  |  |  |
| Migraine > HC |  |  |  |  |  |  |  |
| Calcarine_R (aal) | 8 | -88 | 8 | 435 | 1.679 | 0.175 | 0.000519 |
| Occipital_Inf_L (aal) | -18 | -102 | -8 | 252 | 1.594 | 0.168 | 0.001198 |
| Postcentral_L (aal) | -40 | -24 | 56 | 142 | 1.674 | 0.175 | 0.000548 |
| Precuneus_R (aal) | 10 | -42 | 8 | 56 | 1.656 | 0.179 | 0.000657 |
| Cerebelum_4_5_R (aal) | 8 | -56 | -4 | 46 | 1.640 | 0.171 | 0.000789 |
| Lingual_R (aal) | 26 | -46 | -4 | 44 | 1.576 | 0.169 | 0.000789 |
| Migraine < HC |  |  |  |  |  |  |  |
| SurpraMarginal_R (aal) | 50 | -30 | 46 | 258 | -1.471 | -0.223 | 0.001978 |
| Frontal_Mid_L (aal) | -26 | 52 | 20 | 134 | -1.485 | -0.229 | 0.001818 |
| Frontal_Inf_Orb_R (aal) | 40 | 32 | -10 | 107 | -1.424 | -0.185 | 0.002630 |
| Occipital_Sup_R (aal) | 26 | -66 | 44 | 101 | -1.445 | -0.203 | 0.002322 |
| Frontal_Mid_L (aal) | -30 | 32 | 40 | 53 | -1.496 | -0.156 | 0.001724 |
| Frontal_Inf_Oper_L (aal) | -50 | 18 | 16 | 34 | -1.410 | -0.177 | 0.002845 |

Abbreviation: aal, automated anatomical labeling; HC, healthy controls; MNI, Montreal Neurological Institute; SDM, seed-based d mapping; Calcarine_R, right calcarine gyrus; Occipital_Inf_L, left inferior occipital gyrus; Postcentral_L, left postcentral gyrus; Cerebelum_4_5_R, right cerebellum (lobules IV / V); Lingual_R, right lingual gyrus; SurpraMarginal_R, right supramarginal gyrus; Frontal_Mid_L, left middle frontal gyrus; Frontal_Inf_Orb_R, right inferior frontal gyrus, orbital part; Occipital_Sup_R, right superior occipital gyrus; Frontal_Mid_L, left middle frontal gyrus; Frontal_Inf_Oper_L, left inferior frontal gyrus, opercular part.

Table S7: Clusters showing rs-FC differences in migraine patients compared with HC in subgoup analysis of no-medication status.

| Location | MNI coordinate | | | Cluster  size | SDM-Z  value | Effect size | *p* value |
| --- | --- | --- | --- | --- | --- | --- | --- |
|  | x | y | z |  |  |  |  |
| Migraine > HC |  |  |  |  |  |  |  |
| Precentral_L (aal) | -36 | -22 | 60 | 236 | 1.639 | 0.188 | 0.000551 |
| Temporal_Mid_L (aal) | -52 | 2 | -16 | 46 | 1.511 | 0.173 | 0.002476 |
| Precentral_L (aal) | -24 | -18 | 64 | 30 | 1.525 | 0.177 | 0.002277 |
| Migraine < HC |  |  |  |  |  |  |  |
| Frontal_Mid_L (aal) | -32 | 32 | 40 | 456 | -1.568 | -0.180 | 0.000820 |
| Frontal_Sup_L (aal) | -20 | 26 | 62 | 18 | -1.251 | -0.143 | 0.003875 |

Abbreviation: aal, automated anatomical labeling; HC, healthy controls; MNI, Montreal Neurological Institute; SDM, seed-based d mapping; Precentral_L, left precentral gyrus; Temporal_Mid_L, left middle temporal gyrus; Precentral_L, left precentral gyrus; Frontal_Mid_L, left middle frontal gyrus; Frontal_Sup_L, left superior frontal gyrus, dorsolateral.

Table S8: Clusters showing rs-FC differences in migraine patients compared with HC in subgroup analysis of medication status.

| Location | MNI coordinate | | | Cluster  size | SDM-Z  value | Effect size | *p* value |
| --- | --- | --- | --- | --- | --- | --- | --- |
|  | x | y | z |  |  |  |  |
| Migraine > HC |  |  |  |  |  |  |  |
| Calcarine_L (aal) | -10 | -94 | 2 | 456 | 1.019 | 0.516 | 0.000076 |
| Calcarine_R (aal) | 10 | -94 | 2 | 356 | 1.027 | 0.055 | 0.000008 |
| Lingual_L (aal) | -18 | -54 | 0 | 198 | 1.006 | 0.461 | 0.000502 |
| Migraine < HC |  |  |  |  |  |  |  |
| Supp_Motor_Area_R (aal) | 2 | 20 | 58 | 92 | -1.099 | -0.234 | 0.001902 |
| Frontal_Inf_Oper_R (aal) | 42 | 14 | 30 | 79 | -1.099 | -0.234 | 0.001902 |
| Postcentral_R (aal) | 46 | -28 | 40 | 55 | -1.099 | -0.234 | 0.001902 |
| Occipital_Sup_R (aal) | 32 | -74 | 44 | 41 | -1.099 | -0.234 | 0.001902 |
| Frontal_Inf_Orb_R (aal) | 44 | 30 | -10 | 33 | -1.098 | -0.234 | 0.001902 |
| SupraMarginal_R (aal) | 48 | -40 | 42 | 32 | -1.098 | -0.234 | 0.001902 |
| Frontal_Inf_Oper_L (aal) | -44 | 12 | 22 | 25 | -1.098 | -0.234 | 0.001902 |
| Precentral_L (aal) | -58 | 4 | 20 | 25 | -1.098 | -0.046 | 0.001902 |
| Postcentral_R (aal) | 42 | -28 | 56 | 21 | -1.098 | -0.234 | 0.001902 |
| Angular_R (aal) | 38 | -60 | 52 | 14 | -1.099 | -0.234 | 0.001902 |
| Angular_R (aal) | 38 | -62 | 36 | 13 | -1.099 | -0.234 | 0.001902 |
| Temporal_Inf_R (aal) | 50 | -50 | -24 | 13 | -1.096 | -0.235 | 0.002270 |
| Frontal_Inf_Oper_R (aal) | 44 | 18 | 36 | 13 | -1.098 | -0.234 | 0.002102 |
| Temporal_Mid_R (aal) | 50 | -38 | -14 | 10 | -1.097 | -0.235 | 0.002102 |

Abbreviation: aal, automated anatomical labeling; HC, healthy controls; MNI, Montreal Neurological Institute; SDM, seed-based d mapping; Calcarine_L, left calcarine gyrus; Calcarine_R, right calcarine gyrus; Lingual_L, left lingual gyrus; Supp_Motor_Area_R, right supplementary motor area; Frontal_Inf_Oper_R, right Inferior frontal gyrus, opercular part; Postcentral_R, right postcentral gyrus; Occipital_Sup_R, right superior occipital gyrus; Frontal_Inf_Orb_R , right Iinferior frontal gyrus, orbital part; SupraMarginal_R, right supramarginal gyrus; Frontal_Inf_Oper_L, left inferior frontal gyrus, opercular part; Precentral_L, left precental gyrus; Postcentral_R, right postcentral gyrus; Angular_R, right angular gyrus; Temporal_Inf_R , right inferior temporal gyrus; Frontal_Inf_Oper_R, right inferior frontal gyrus, opercular part; Temporal_Mid_R, right middle temporal gyrus.


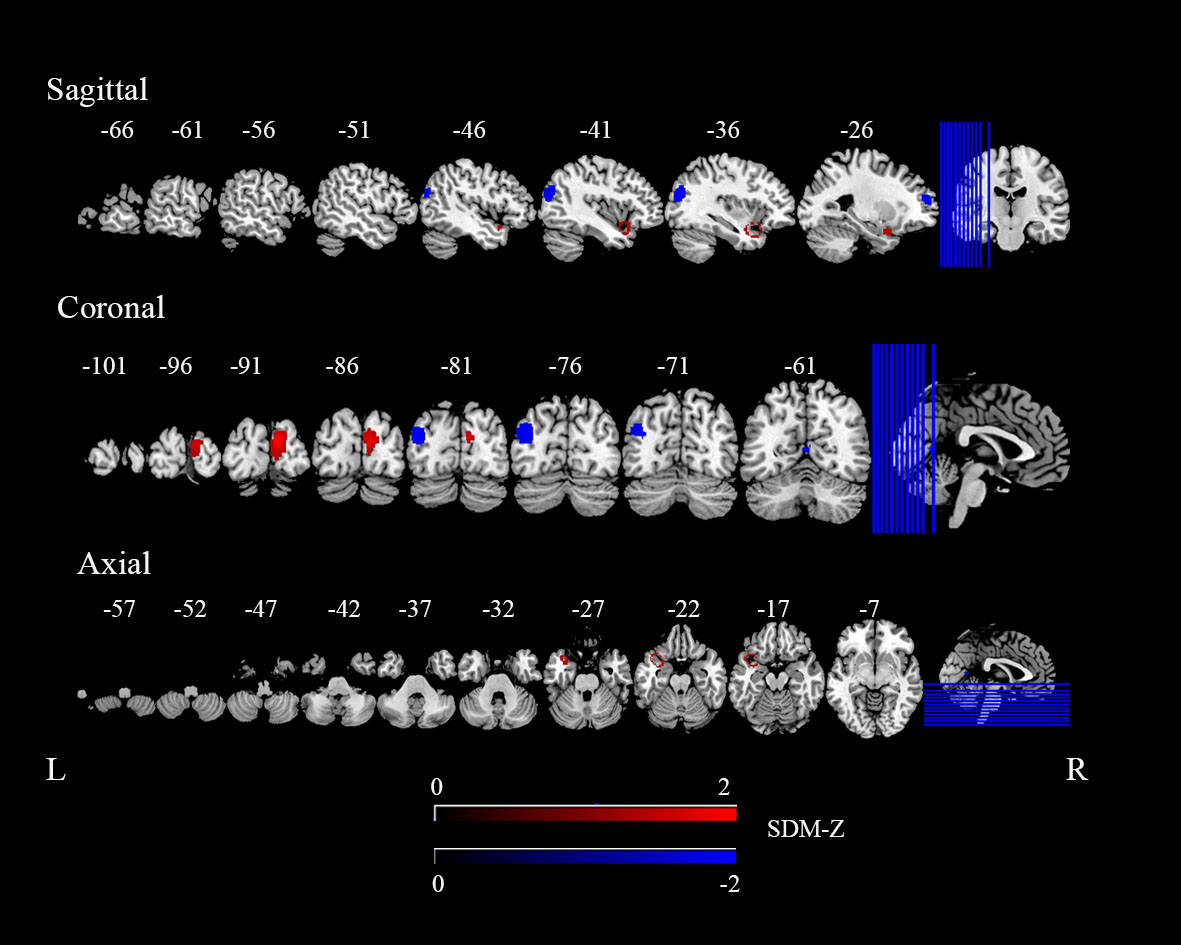


Figure S1: Clusters showing rs-FC differences in migraine patients compared with HC in subgroup analysis of ICA method. The areas of increased (red) and decreased (blue) FC in subgroup analyses of ICA method. “R” and “L” denote the right and left sides of the brain, respectively. The color bar indicates the maximum and minimum SDM-Z value.


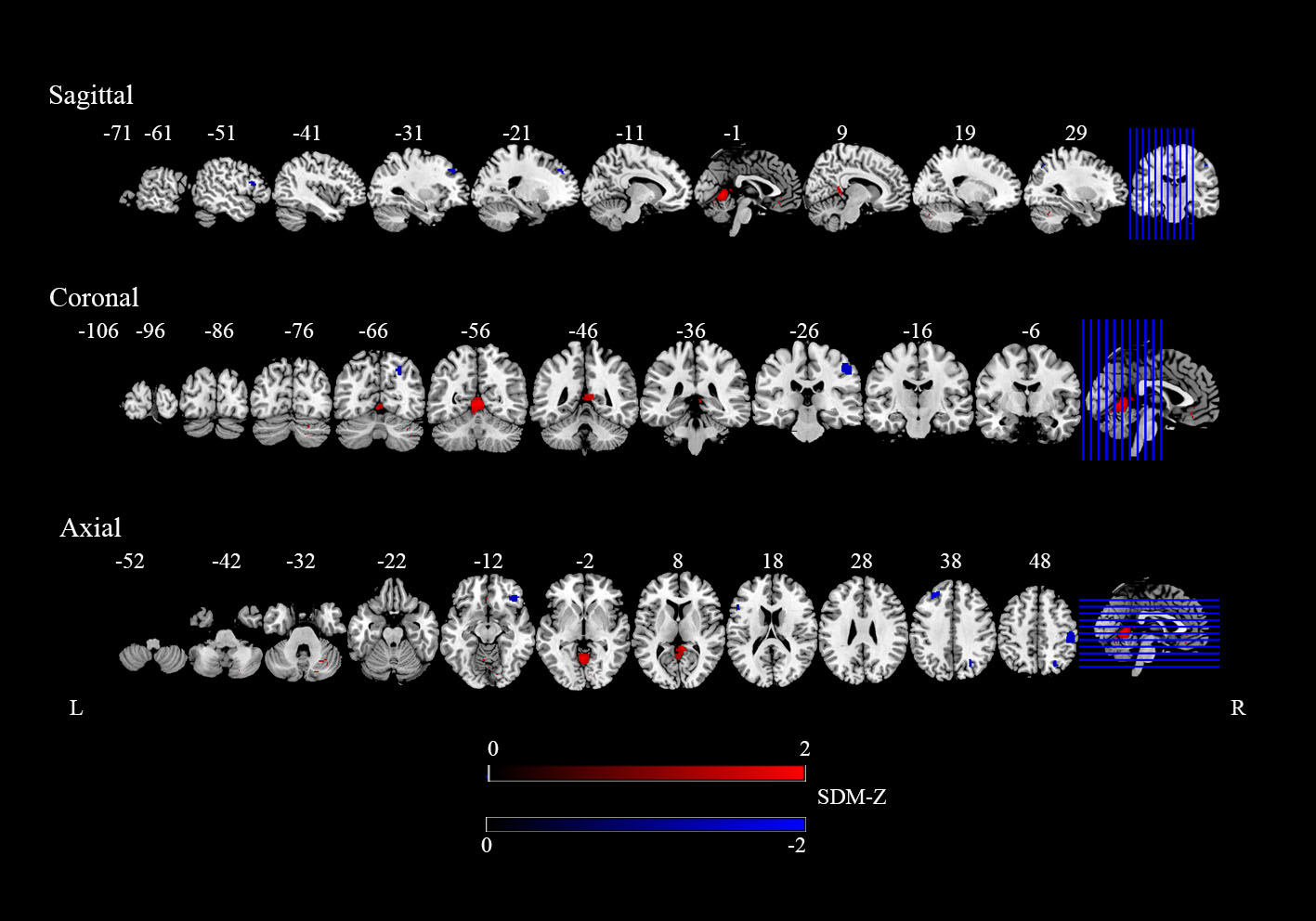


Figure S2: Clusters showing rs-FC differences in migraine patients compared with HC in subgroup analysis of seed-based function connectivity method. The areas of increased (red) and decreased (blue) FC in subgroup analyses of seed-based method. “R” and “L” denote the right and left sides of the brain, respectively. The color bar indicates the maximum and minimum SDM-Z value.


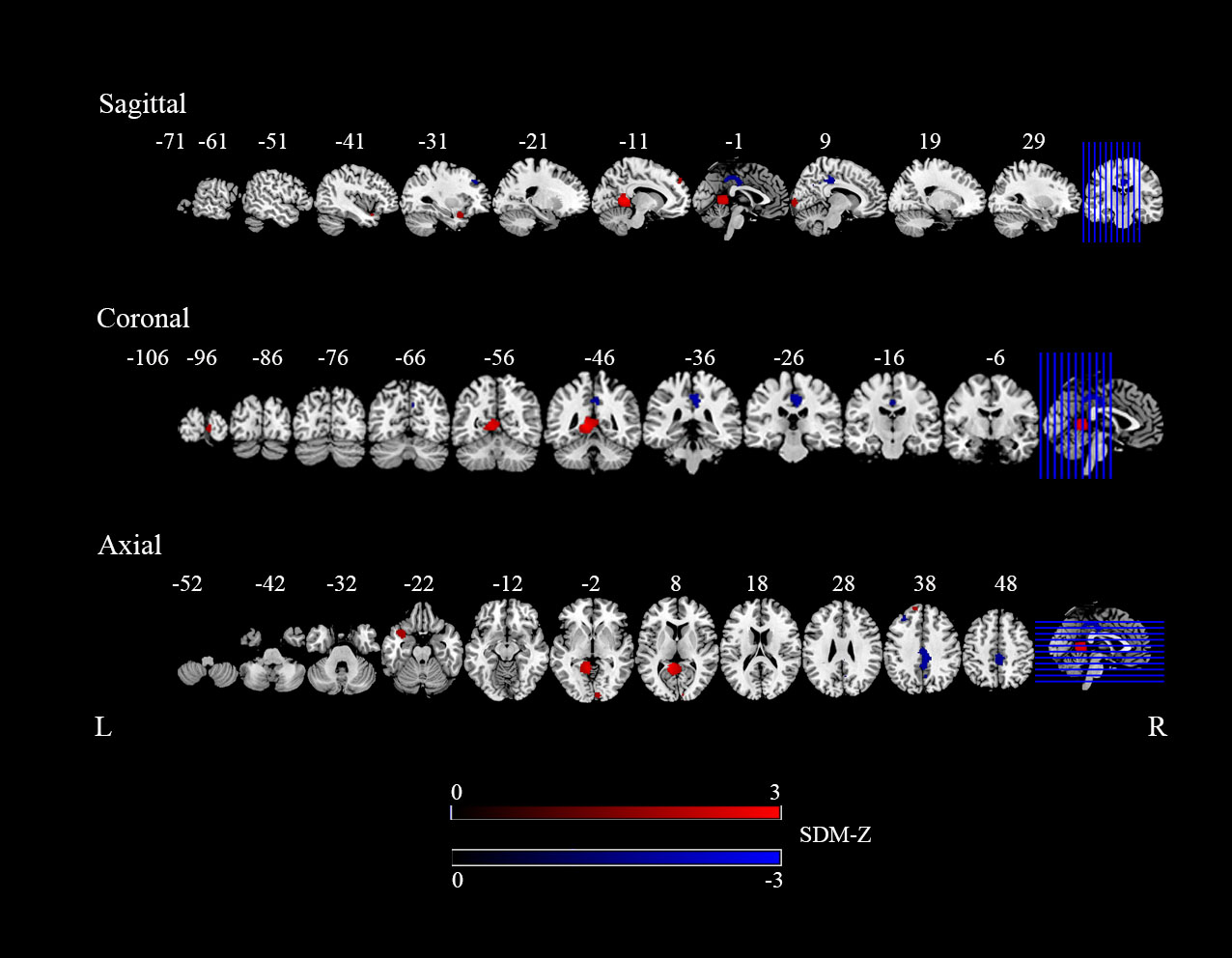


Figure S3: Clusters showing rs-FC differences in migraine patients compared with HC in subgroup analysis of migraine without aura (MowA). The areas of increased (red) and decreased (blue) FC in subgroup analyses of MowA. “R” and “L” denote the right and left sides of the brain, respectively. The color bar indicates the maximum and minimum SDM-Z value.


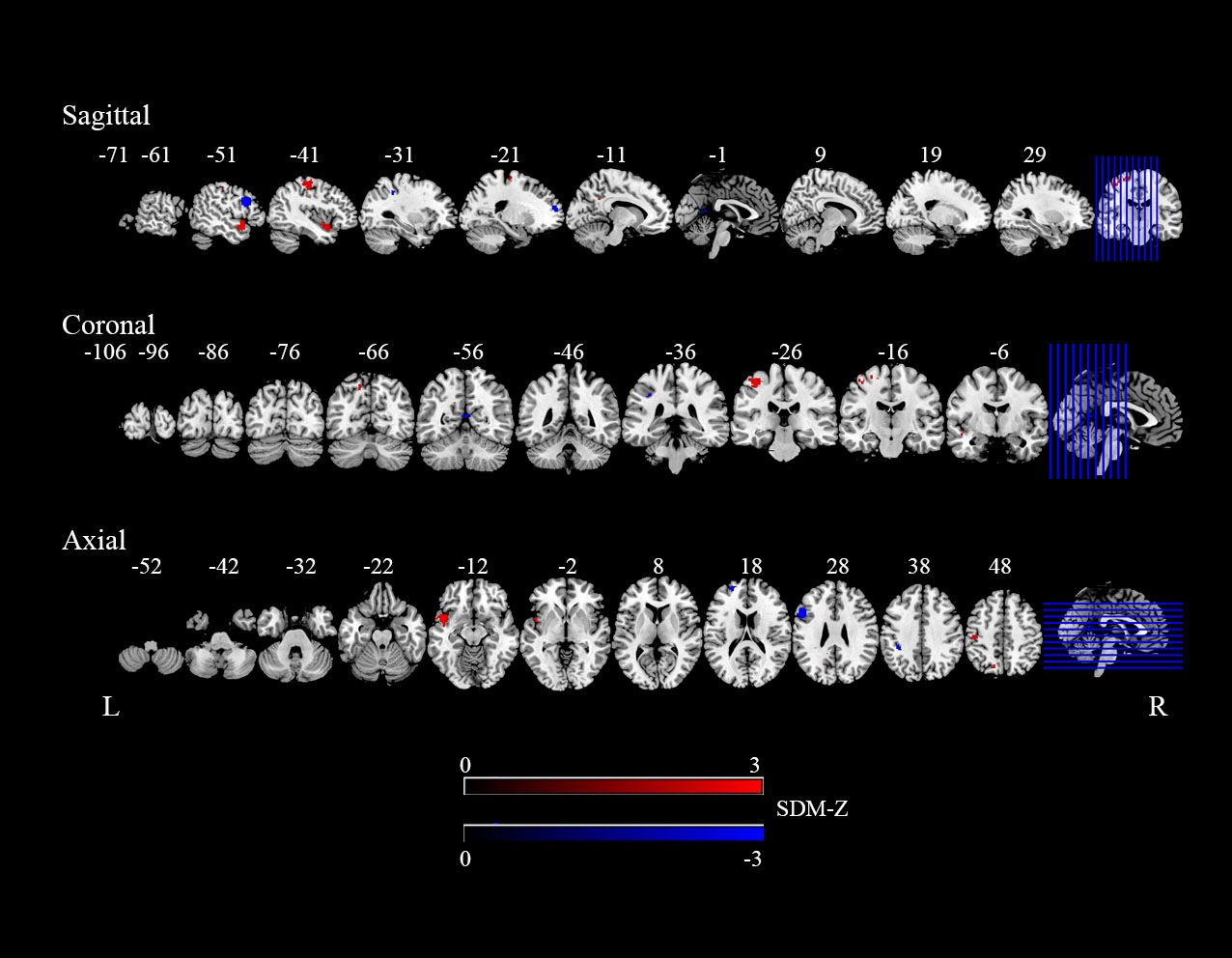


Figure S4: Clusters showing rs-FC differences in migraine patients compared with HC in subgroup analysis of migraine (MIG). The areas of increased (red) and decreased (blue) FC in subgroup analyses of migraine. “R” and “L” denote the right and left sides of the brain, respectively. The color bar indicates the maximum and minimum SDM-Z value.


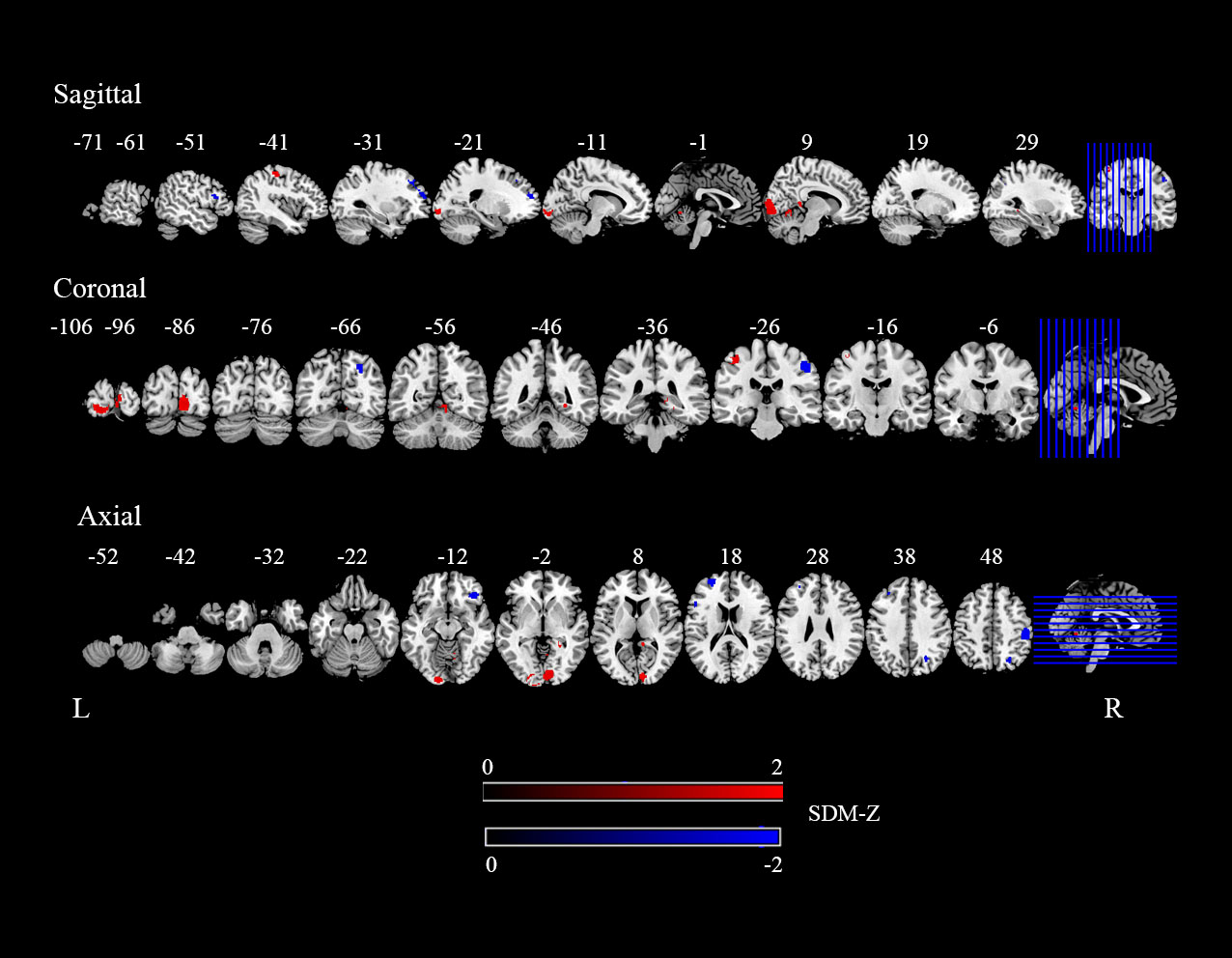


Figure S5: Clusters showing rs-FC differences in migraine patients compared with HC in subgroup analysis of adults group. The areas of increased (red) and decreased (blue) FC in subgroup analyses of adult group. “R” and “L” denote the right and left sides of the brain, respectively. The color bar indicates the maximum and minimum SDM-Z value.


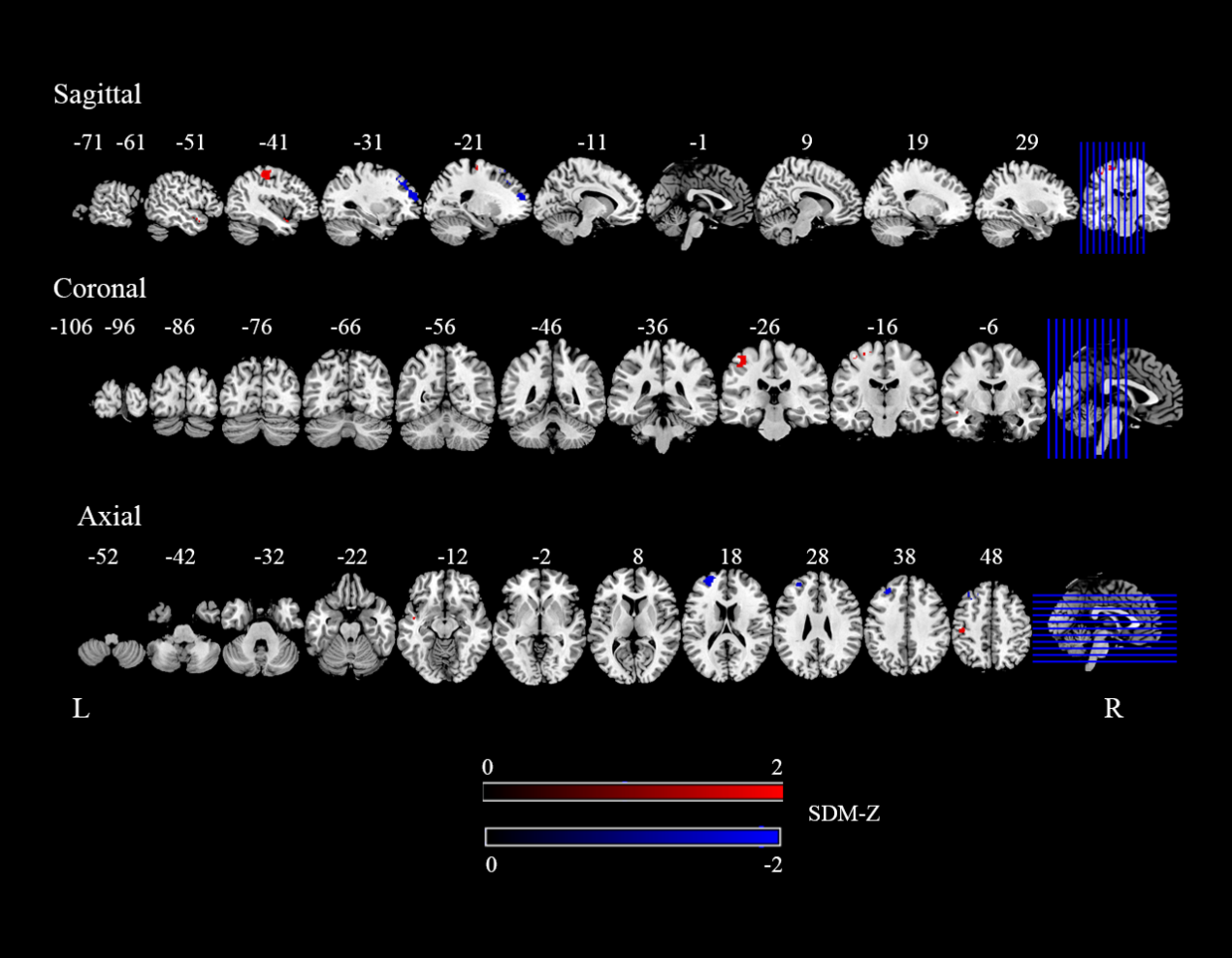


Figure S6: Clusters showing rs-FC differences in migraine patients compared with HC in subgroup analysis of no-medication status. The areas of increased (red) and decreased (blue) FC in subgroup analyses of no-medication. “R” and “L” denote the right and left sides of the brain, respectively. The color bar indicates the maximum and minimum SDM-Z value.


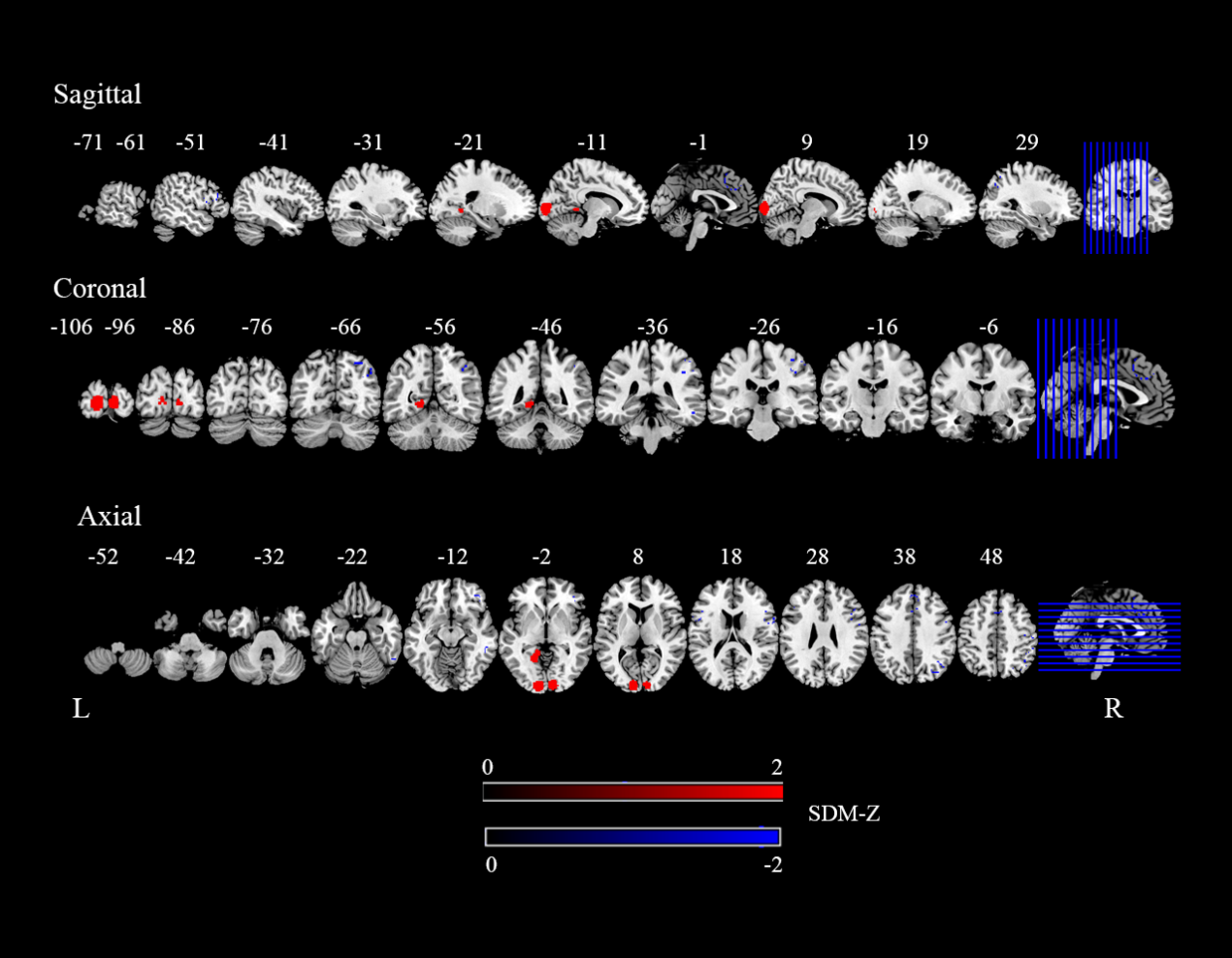


Figure S7: Clusters showing rs-FC differences in migraine patients compared with HC in subgroup analyses of medication status. The areas of increased (red) and decreased (blue) FC in subgroup analyses of medication status. “R” and “L” denote the right and left sides of the brain, respectively. The color bar indicates the maximum and minimum SDM-Z value.


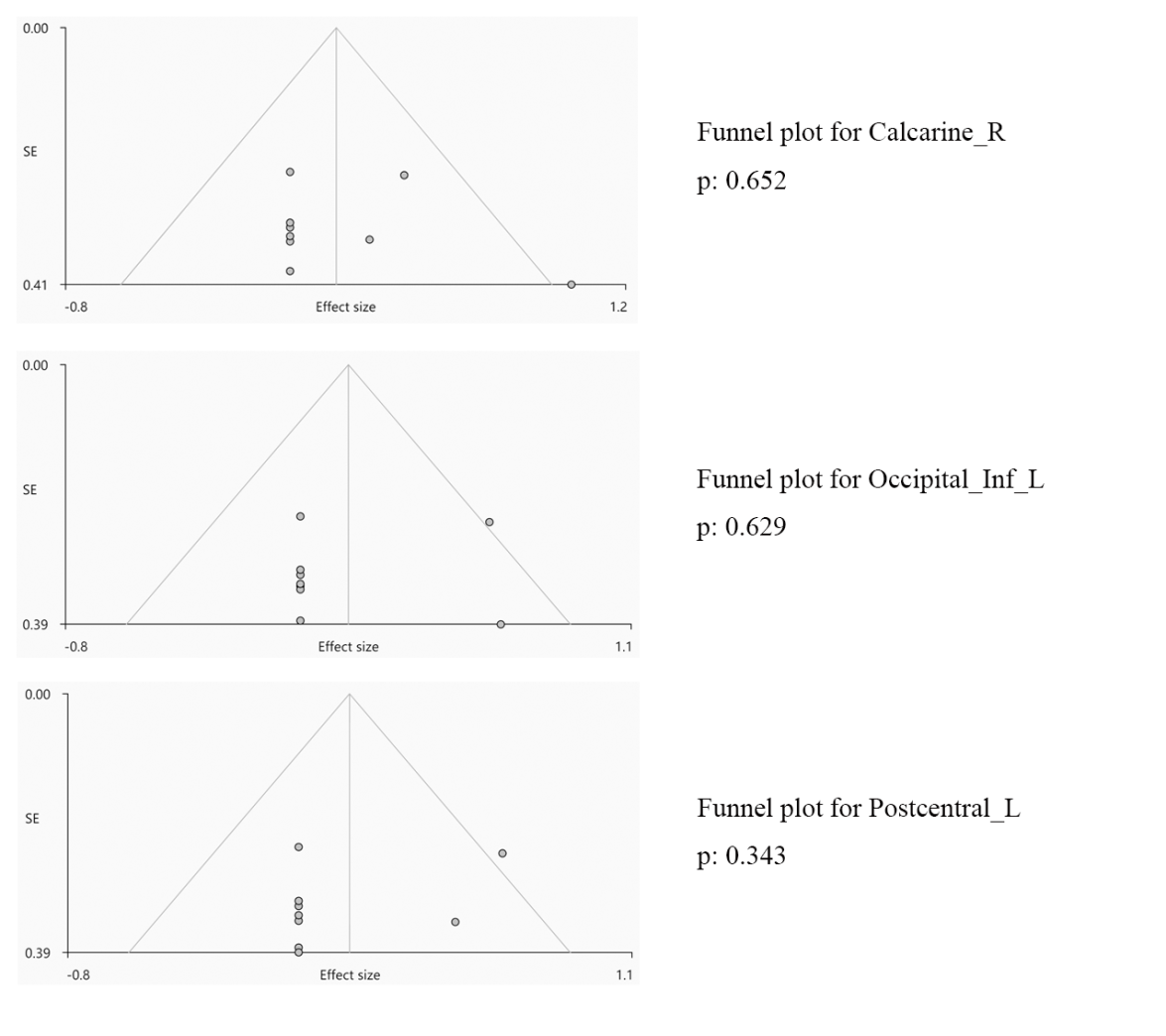


Figure S8: **A** funnel plot of the meta-analysis to visualize the possibility of publication bias. Abbreviation: Calcarine_R, right calcarine gyrus; Occipital_Inf_L, left inferior occipital gyrus; Postcentral_L, left postcentral gyrus.


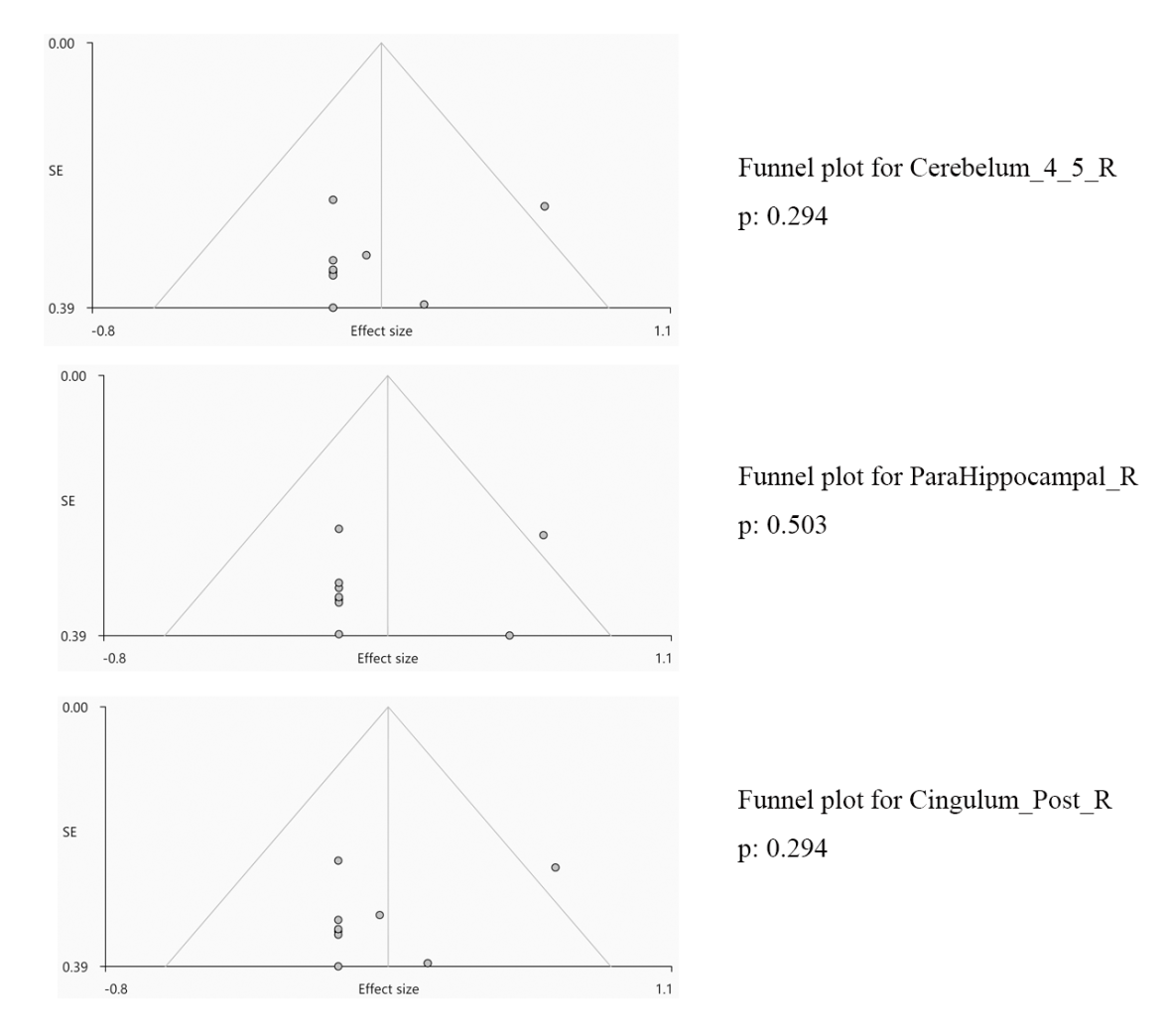


Figure S9: A funnel plot of the meta-analysis to visualize the possibility of publication bias. Abbreviation: Cerebelum_4_5_R, right cerebellum (lobules IV / V); ParaHippocampal_R, right parahippocampal gyrus; Cingulum_Post_R, right posterior cingulate gyrus.


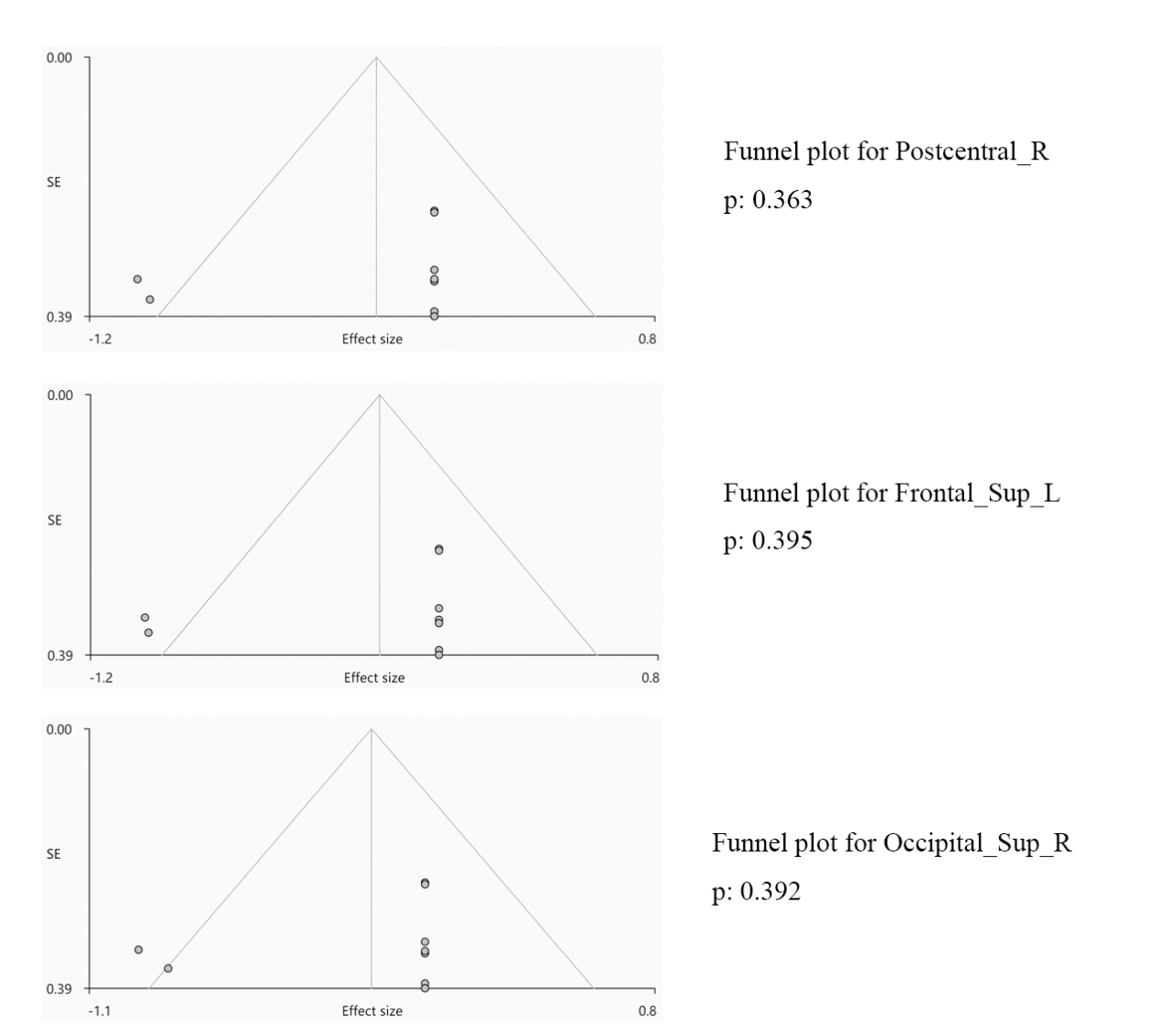


Figure S10: A funnel plot of the meta-analysis to visualize the possibility of publication bias. Abbreviation: Postcentral_R, right postcentral gyrus; Frontal_Sup_L, left superior frontal gyrus; Occipital_Sup_R, right superior occipital gyrus.


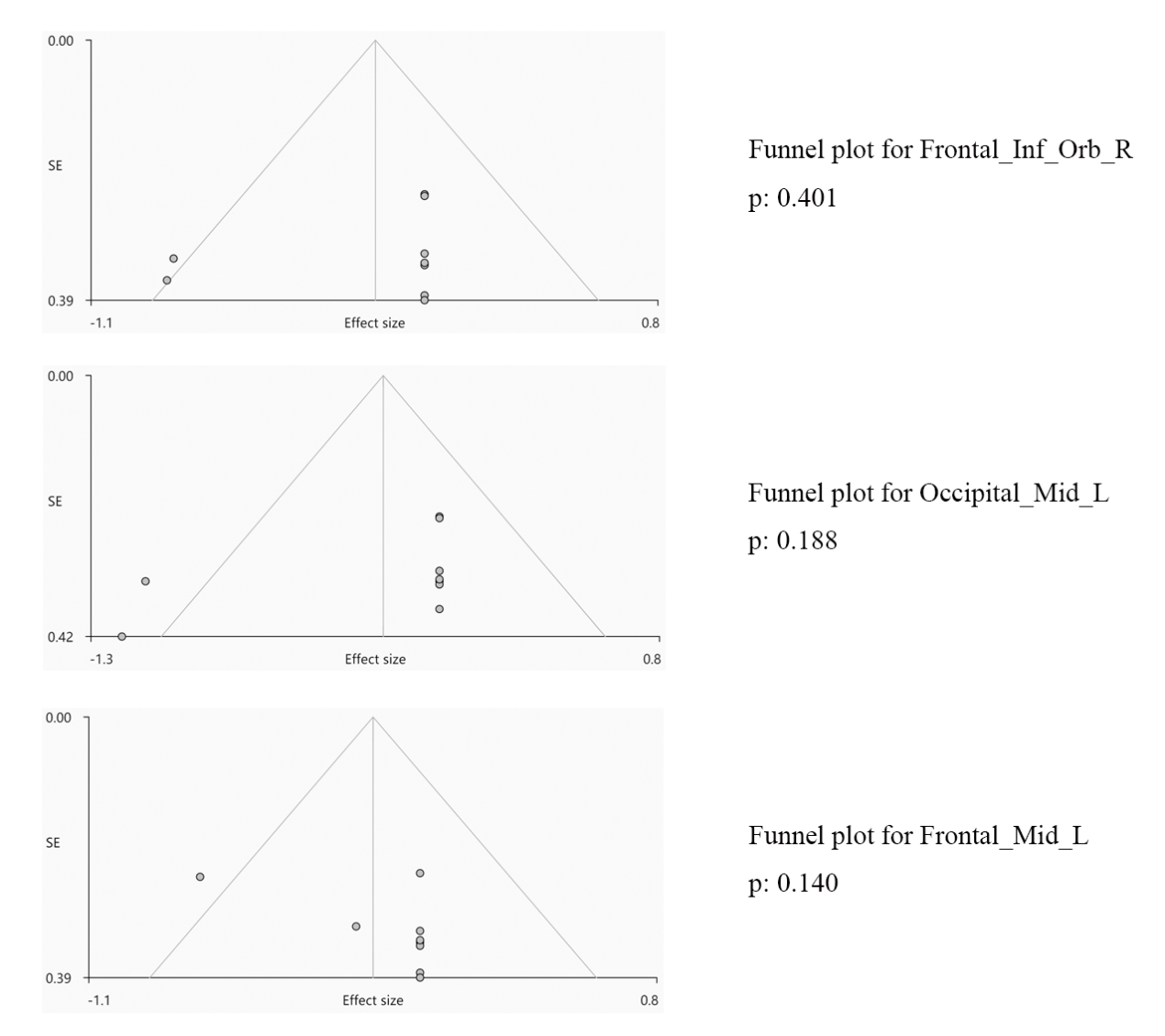


Figure S11: **A** funnel plot of the meta-analysis to visualize the possibility of publication bias. Abbreviation: Frontal_Inf_Orb_R, right orbital inferior frontal gyrus; Occipital_Mid_L, left middle occipital gyrus; Frontal_Mid_L, left middle frontal gyrus.


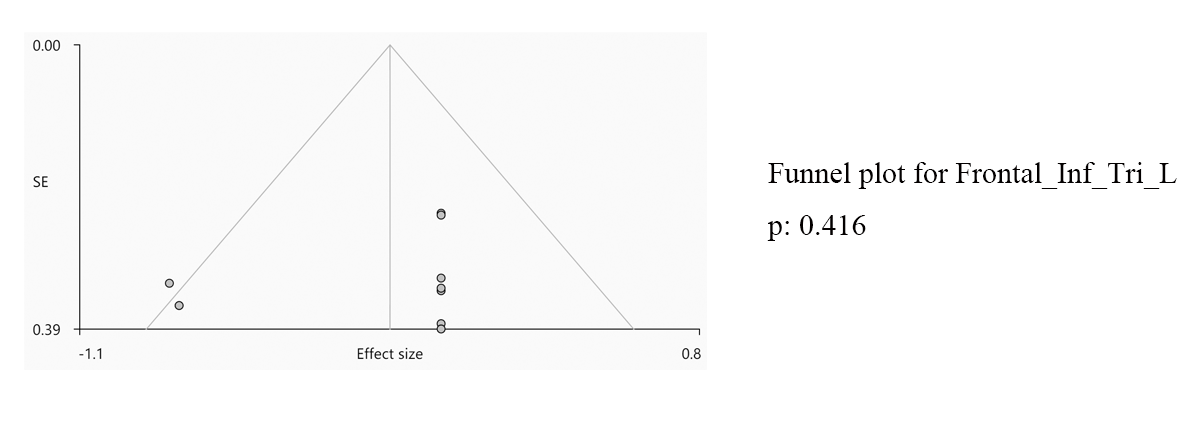


Figure S11: **A** funnel plot of the meta-analysis to visualize the possibility of publication bias. Abbreviation: Frontal_Inf_Tri_L, left inferior frontal gyrus triangular part.
